# COVID-19 health worries and anxiety symptoms among older adults: the moderating role of ageism

**DOI:** 10.1017/S1041610220001258

**Published:** 2020-06-17

**Authors:** Yoav S. Bergman, Sara Cohen-Fridel, Amit Shrira, Ehud Bodner, Yuval Palgi

**Affiliations:** 1 Faculty of Social Work, Ashkelon Academic College, Ashkelon, Israel; 2 School of Education, Bar-Ilan University, Ramat-Gan, Israel; 3 Interdisciplinary Department of Social Sciences, Bar-Ilan University, Ramat-Gan, Israel; 4 Department of Music, Bar-Ilan University, Ramat-Gan, Israel; 5 Department of Gerontology, University of Haifa, Haifa, Israel

**Keywords:** ageism, anxiety symptoms, COVID-19, COVID-19-related health worries

## Abstract

A prominent feature of anxiety in late life is concerns regarding physical health. Anxiety symptoms among older adults have been connected with various psychological outcomes, including social isolation and loneliness. During the coronavirus disease 2019 (COVID-19) pandemic, many societies have demonstrated increased ageist attitudes, encouraging older adults to distance themselves from society. Accordingly, the current study examined the moderating role of COVID-19-related ageism in the connection between COVID-19 health worries and anxiety symptoms among older adults. Data were collected from 243 older adults (age range 60–92; *M* = 69.75, *SD* = 6.69), who completed scales assessing COVID-19-related health worries and ageism, as well as anxiety symptoms. The results demonstrated that both health worries and ageism were positively associated with anxiety symptoms. Moreover, the connection between health worries and anxiety symptoms was more pronounced among older adults with high ageism levels. The study highlights the vulnerability of older adults in general, and ageist older adults in particular, to the negative consequences of COVID-19-related health worries, and emphasizes the role of the increased ageist stance of society during the pandemic in this regard.

## Introduction

Anxiety symptoms and anxiety disorders constitute a major clinical issue in late life. The prevalence rate of anxiety disorders ranges from 1.2% to 15% among older adult populations (Byrne and Pachana, 2011) and is undoubtedly higher for anxiety symptoms which do not reach the diagnostic cutoff point. Late-life anxiety has been associated with negative psychological outcomes, including loneliness and social isolation (Fees *et al.*, 1999).

A prominent characteristic of anxiety symptoms, which also constitutes a diagnostic criterion for general anxiety disorder (American Psychiatric Association, 2013), is excessive and uncontrollable worry. While the causal connection between worries and anxiety is open to debate (see Judah *et al.*, 2013), it is nevertheless suggested that the cognitive phenomenon of worry is central to the anxious experience and that a major feature of anxiety-related worries among older adults is concerns regarding physical health (Diefenbach *et al.*, 2001). These concerns may be exacerbated by the coronavirus disease 2019 (COVID-19) pandemic, which constitutes a particular threat to anxiety symptoms among older adults (Girdhar *et al.*, 2020).

According to Ayalon (2020), the COVID-19 pandemic has been portrayed, from its initial stages, as a “problem of older adults,” and the portrayal of older adults as a homogenous group which is particularly vulnerable to the Coronavirus, sometimes even by government officials, has resulted in increased ageism around the world. Accordingly, older adults are increasingly encouraged by society to isolate themselves from younger people, thereby creating an age distinction in the perception of the consequences of COVID-19. This policy may support ageist beliefs regarding the need to limit intergenerational encounters and to impede the tendency of older adults to infiltrate into the identity of younger people (North and Fiske, 2013). Additionally, according to Levy’s (2009) Stereotype Embodiment Theory, older adults may internalize their ageist experiences and attitudes, which become self-definitions through which they see themselves and perceive their self-image (Levy, 2009). Accordingly, as the need for social distancing is rationalized by the older adults’ physical susceptibility to the Coronavirus, ageist older adults may be more susceptible to the negative psychological consequences of COVID-19 health-related worries, since such worries could implicitly link them with the social group of “older adults,” which they avoid and devalue.

Accordingly, the current study aimed at examining the role of ageism in the connection between COVID-19 health worries and anxiety symptoms among older adults and explored the moderating role of ageism as strengthening this connection. We hypothesized that both COVID-19 health worries and COVID-19-related ageism would be positively related with anxiety symptoms. Moreover, we hypothesized that the health worries—anxiety symptoms connection—would be stronger among older adults who display high levels of ageism.

## Methods

### Participants and procedure

Data were collected from 243 Jewish Israelis and between March 16 and April 14, 2020. On the last day of collection, 12,361 people in Israel had tested positive for COVID-19, and 123 had died. Age ranged from 60 to 92 (*M* = 69.75, *SD* = 6.69), and 75 participants (30.9%) were men. Most of the participants (*n* = 177, 72.8%) reported that they were in a relationship. Two participants (0.8%) had no formal education, 9 (3.7%) had some high-school education, 17 (7.0%) had a full high-school education, 35 (14.4%) had some undergraduate courses, and 179 (73.2%) had an academic degree. The majority of participants rated their economic status and health status as “pretty good” or above (*n* = 220, 90.2%; *n* = 216, 89.2%, respectively). The online questionnaire was distributed across multiple social media. Anonymity was guaranteed as identifying details were neither required nor requested. The study scales were back-translated into Hebrew by two experienced bilingual psychologists. The study received ethical approval by the institutional review board of [BLINDED FOR REVIEW].

### Materials

Participants completed background characteristics, including age, gender, marital status, education (rated from 1, “without formal education” to 6, “formal university degree”), and economic and health status (rated from 1, “not good at all” to 5, “very good”). We also examined exposure to COVID-19, calculated as the sum score of six different types of exposure, and behavioral changes due to the pandemic, calculated as the sum of 11 changes in the individual’s behaviors. Both exposure and behavior changes were dichotomous (0 = “no,” 1 = “yes”; for details, see Table S1 published as supplementary material online attached to the electronic version of this paper).

Participants completed the following measures while referring directly to feelings and symptoms they experienced since the COVID-19 outbreak. For means, SDs, and correlation matrix for the study variables, see Table S2 published as supplementary material online attached to the electronic version of this paper.

COVID-19 worries were examined with four items, rated on a scale ranging from 1 (“not concerned at all”) to 5 (“extremely concerned”). A mean score was calculated, and higher scores reflect higher levels of health concerns. Cronbach’s alpha was .83 (for details regarding the items used to assess COVID-19 worries and ageism, see Appendix A1 published as supplementary material online attached to the electronic version of this paper).

Ageism was assessed with seven items taken from North and Fiske’s (2013) scale, which were adapted to examine ageist attitudes during the coronavirus pandemic. We chose to focus on items pertaining to fear of intergenerational segregation and social identity infiltration (North and Fiske, 2013) due to our wish to keep the study questionnaires brief on the one hand, while keeping them relevant to COVID-19 concerns on the other hand. Participants rated their responses a scale ranging from 1 (“strongly disagree”) to 6 (“strongly agree”). A mean score was calculated, and higher scores reflect higher levels of ageism. This scale has demonstrated strong internal consistency (North and Fiske, 2013), and in the current study, Cronbach’s *α* was .76.

Anxiety symptoms were assessed with the seven-item Generalized Anxiety Disorder scale (Spitzer *et al.*, 2006). Participants rated their symptoms (e.g. “feeling nervous, anxious, or on edge”) during the last two weeks on a four-point scale (0=“not at all” to 3=“almost every day”). Ratings were summed with higher scores reflecting increased anxiety. Internal reliability was good (Cronbach’s *α* = .89).

### Data analysis

Analyses were conducted using the SPSS-25 software (IBM), and significant interactions were probed using Model 1 of the PROCESS 3.4 macro (Hayes, 2018), which calculates the regression coefficients for the effects of the predictor (i.e. COVID-19 worries) on the predicted (i.e. anxiety symptoms) variable for both ±1 SD of the moderator (i.e. ageism). A hierarchical regression was conducted in order to examine the study hypotheses, with anxiety symptoms as the predicted variable. The first step included the socio-demographic and covariate factors of participants’ age, gender, relationship status, education level, economic status, self-rated health, COVID-19 exposure, and COVID-19 behavioral changes. The second step included COVID-19 worries and ageism, and the third and final step included the COVID-19 worries × ageism interaction (see Table 1 for regression coefficients). A power analysis for detecting a medium to strong effect size (0.20) with 11 predictors required a sample size of 178, indicating that the current sample was sufficient for examining the study model. Potential multicollinearity between the predicting variables was rejected, as the values of both tolerance and variance inflation factor for the study variables ranged between 0.71 and 0.96, and between 1.04 and 1.39, respectively.

**Table 1.** Standard multiple regression predicting anxiety symptoms

Predictor	Δ*R*^2^	*B*	*SE*	LLCI^a^	ULCI^a^	*β*	*t*	*p*
Step 1	.118***							
Age		−.01	.04	−.08	.07	−.01	−.14	.891
Gender^b^		2.07	.56	.96	3.18	.25	3.67	<.001
Relationship status^c^		.96	.58	−.19	2.11	.11	1.66	.099
Education		−1.04	.29	−1.62	−.46	−.23	−3.56	<.001
Economic status		−.26	.33	−.92	.40	−.06	−.77	.439
Self-rated health		−.15	.31	−.76	.46	−.04	−.49	.622
COVID-19 exposure		.17	.23	−.29	.63	.05	.72	.473
COVID-19 behavioral changes		.16	.11	−.06	.39	.09	1.43	.155
Step 2	.122***							
COVID-19 health worries		1.55	.27	1.02	2.08	.36	5.78	<.001
Ageism		1.01	.47	.08	1.94	.13	2.14	.033
Step 3	.019*							
COVID-19 health worries × ageism		.52	.22	.09	.95	.15	2.38	.018
Total *R*²	.259							
*N*	243							

a Lower/upper limits for 95% confidence interval for *B* values.

b Gender: 0 = Male, 1 = Female.

c Relationship status: 0 = not in a relationship; 1 = in a relationship.

* = *p* < .05, *** = *p* < .001.

## Results

**Figure 1.**
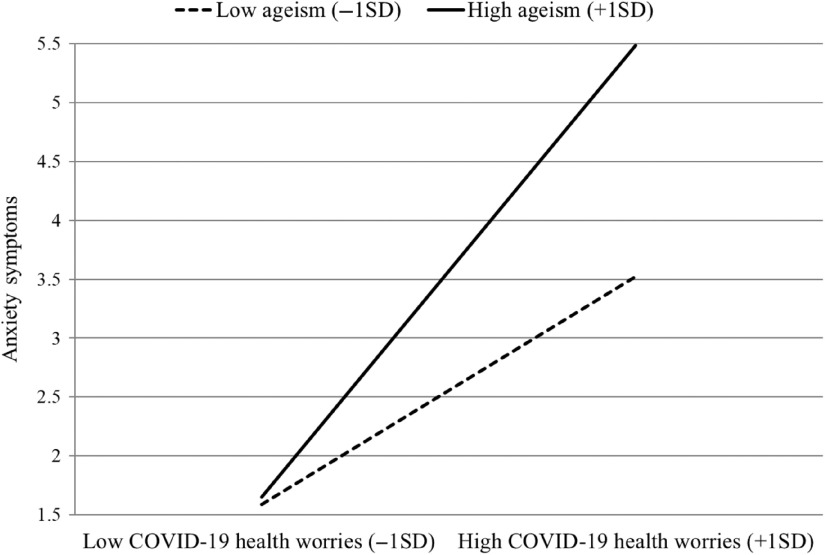
The two-way interaction between COVID-19 health worries and ageism in predicting anxiety symptoms.

As Table 1 demonstrates, COVID-19 health worries (*B* = 1.55, *SE* = .27, *β* = .36, *p* < .001) and ageism (*B* = 1.01, *SE* = .47, *β* = .13, *p* < .05) were positively related with anxiety symptoms, which is in line with the first hypothesis. The second hypothesis was also confirmed, as a significant interaction COVID-19 health worries × ageism was discovered (*B* = .52, *SE* = .22, β = .15, *p* < .05), accounting for an additional 1.9% of variance (total *R²* = .259). Upon probing the interaction with PROCESS (Hayes, 2018), we discovered that while the association between health worries and anxiety symptoms remained significant for individuals with both low and high ageism levels, this association was significantly stronger among individuals with high ageism (*B* = 2.13, *SE* = .36, *β* = .49, *p* < .001) in comparison to those with low ageism (*B* = 1.07, *SE* = .33, *β* = .25, *p* < .01; see Figure 1). The results remained unchanged when the study model was examined without the covariates.

## Discussion

Anxiety symptoms among older adults are often characterized by health-related concerns. These concerns may be exacerbated due to the current COVID-19 pandemic, and by increased ageist views following the pandemic, demonstrated by the social distancing policy aimed at older adults. Our results demonstrate that both COVID-19 health-related worries and ageism resulting from the current pandemic were positively associated with anxiety symptoms among older adults. Moreover, the connection between COVID-19-related health worries and anxiety symptoms was stronger among individuals with high levels of ageism. In fact, while the mean difference in anxiety symptoms between individuals with high worries and low/high ageism (*M* = 3.61 vs. 5.47, respectively) seems modest, it is quite important, when one takes into account that the cutoff point for mild anxiety is 5 (Spitzer *et al.*, 2006), indicating that older adults with high ageism and COVID-19 worries meet the clinical threshold for mild anxiety.

In line with the Stereotype Embodiment Theory (Levy, 2009), older adults with high levels of ageism (i.e. self-ageist older adults) may perceive their health both reduced and prone to threats and may therefore be at a higher risk for displaying a stronger connection between the COVID-19 health concerns and anxiety symptoms. This could also stem from the fact that such individuals’ anxiety is exacerbated, since they experience COVID-19 worries in connection with the social group of “older adults,” toward which they are reluctant. Moreover, in light of the importance of meaningful social relationships for older adults (Carstensen *et al.*, 1999), it is possible that ageist older adults may be particularly at risk for negative psychological consequences during the pandemic. While the current study did not examine older adults’ perceptions of the COVID-19 physical age-related separation restrictions, encouraged at times not only by society but by government officials (Ayalon, 2020), future studies can examine the additive value of such perceptions to the explained variance of anxiety symptoms.

Several limitations should be mentioned. First, the cross-sectional nature of the data precludes the ability to establish causality. Namely, it is not clear whether COVID-19 worries produce anxiety symptoms, or vice versa. Accordingly, longitudinal designs are needed to further validate our findings. Second, due to the online nature of the study, it is possible that older adults who are less computer-proficient were precluded from taking part in the study, and it is important to examine the results using additional data gathering methods. Moreover, while we assumed that older individuals who participate in an online study would not demonstrate significant cognitive difficulties, we did not examine this issue. Additionally, we assessed ageism with an adapted short version of North and Fiske’s scale, and examining additional aspects of ageism (e.g. consumption; see North and Fiske, 2013) may provide a deeper understanding of its role regarding health concerns and anxiety symptoms. Finally, we did not assess pre-pandemic health worries, anxiety symptoms, and ageism and therefore could not control for possible changes in the levels of all three variables among the participants.

Nevertheless, our study highlights how the rise in ageism during the COVID-19 pandemic poses additional difficulties for older adults, beyond the understandable concerns and uncertainty we all face. While research has addressed the effect of COVID-19 on older adults (e.g. Lopez *et al.*, 2020), to the best of our knowledge, this is the first attempt to examine the connection between health worries, anxiety symptoms, and ageism in the context of the pandemic. There is little doubt that physical distancing constitutes a key factor in controlling the outbreak of COVID-19. However, a continuous effort is required to understand the social implications of this phenomenon on older adults, which may not only result in increased worry, but in increased anxiety and psychological distress.

## Supplementary material

For supplementary material accompanying this paper visit http://dx.doi.org/10.1017/S1041610220001258. click here to view supplementary material
