# Supplementary material for: COVID-19 health worries and anxiety symptoms among older adults: the moderating role of ageism
Source: Int Psychogeriatr. 2020 Jun 17:1–5. doi: 10.1017/S1041610220001258 (PMC7348214; doi:10.1017/S1041610220001258)
Supplement: Supplementary file 1 [file S1041610220001258sup.zip › S1041610220001258supp001.docx]

| Appendix A1. Items used for examining COVID-19 health worries and ageism | | |
| --- | --- | --- |
|  |  |  |
| **COVID-19 health worries** | | |
| 1. I am worried that I will be infected by the virus |  |  |
| 1. I am worried that one of the people close to me will be infected by the virus |  |  |
| 1. I am worried that one of my family members will be infected by the virus |  |  |
| 1. I am worried that I am a carrier of the virus and might transmit it to my close ones |  |  |
| **Ageism** | | |
| 1. Doctors spend too much time treating sickly older people |  |  |
| 1. Older people are too big a burden on the healthcare system |  |  |
| 1. Older people are often too much of a burden on families |  |  |
| 1. Older people should be blamed for the fact that due to the fear that they will be infected in public transportation, public transportation will cease to operate for younger people |  |  |
| 1. Without the fear of infecting older people, all the calamity surrounding the Coronavirus would not have occurred to such an extent |  |  |
| 1. Older people should be blamed for the fear of infection, since now, younger people cannot hang out in public places |  |  |
| 1. The government shouldn’t close down the country for such a small number of people, who don’t have that long to live anyways |  |  |
|  | | |
