# Supplementary material for: COVID-19 health worries and anxiety symptoms among older adults: the moderating role of ageism
Source: Int Psychogeriatr. 2020 Jun 17:1–5. doi: 10.1017/S1041610220001258 (PMC7348214; doi:10.1017/S1041610220001258)
Supplement: Supplementary file 1 [file S1041610220001258sup.zip › S1041610220001258supp002.docx]

| Table S1. Frequencies for COVID-19 exposure and behavioral changes | | |
| --- | --- | --- |
|  | n | % |
| **COVID-19 exposure** | | |
| Being tested positive for the coronavirus | 3 | 1.2 |
| Being (or having been) in self-isolation | 30 | 12.3 |
| Knowing family members who were tested positive | 31 | 12.8 |
| Knowing family members who are (or were) in self-isolation | 93 | 38.3 |
| Knowing people in self-isolation | 137 | 56.4 |
| **COVID-19 behavioral changes** | | |
| Avoid shaking hands | 195 | 80.2 |
| Avoiding hugs | 204 | 84.0 |
| Keeping physical distance from others | 215 | 88.5 |
| Avoiding social events | 218 | 89.7 |
| Going out less frequently | 224 | 92.2 |
| Avoid inviting or meeting with people | 211 | 86.8 |
| Using mask or gloves | 111 | 45.7 |
| Avoid going to public places | 211 | 86.8 |
| Washing hands more often | 221 | 90.9 |
| Buying more food and water than usual | 81 | 33.3 |
| Canceling/changing significant plans | 145 | 59.7 |
| *Note. N*=243. | | |
