# Supplementary material for: COVID-19 health worries and anxiety symptoms among older adults: the moderating role of ageism
Source: Int Psychogeriatr. 2020 Jun 17:1–5. doi: 10.1017/S1041610220001258 (PMC7348214; doi:10.1017/S1041610220001258)
Supplement: Supplementary file 1 [file S1041610220001258sup.zip › S1041610220001258supp003.docx]

Table S2

Means, SDs, and Pearson correlations among study variables

|  | Variable | M / % | SD | 1 | 2 | 3 | 4 | 5 | 6 | 7 | 8 | 9 | 10 |
| --- | --- | --- | --- | --- | --- | --- | --- | --- | --- | --- | --- | --- | --- |
| 1. | Age | 69.75 | 6.69 | -- |  |  |  |  |  |  |  |  |  |
| 2. | Gender^a^ (male) | 30.9% |  | -.16* | -- |  |  |  |  |  |  |  |  |
| 3. | Relationship status^b^ (not in a relationship) | 27.2% |  | -.07 | -.25*** | -- |  |  |  |  |  |  |  |
| 4. | Education | 5.56 | .88 | -.11 | .08 | .06 | -- |  |  |  |  |  |  |
| 5. | Economic status | 3.68 | .86 | -.09 | .03 | .17** | .28*** | -- |  |  |  |  |  |
| 6. | Self-rated health | 3.71 | .92 | -.18** | .21** | -.02 | .21** | .45*** | -- |  |  |  |  |
| 7. | COVID-19 exposure | 1.28 | 1.06 | -.12 | .03 | .04 | -.01 | .09 | .09 | -- |  |  |  |
| 8. | COVID-19 behavioral changes | 8.38 | 2.16 | -.03 | -.04 | .06 | .01 | .02 | -.06 | .05 | -- |  |  |
| 9. | COVID-19 worries | 3.01 | .89 | -.09 | -.05 | .04 | -.16* | -.17** | -.19** | .02 | .18** | -- |  |
| 10. | Ageism | 1.44 | .51 | .12 | .04 | -.12 | -.22** | -.02 | -.08 | .06 | -.06 | -.06 |  |
| 11. | Anxiety | 3.01 | 3.89 | -.02 | .19** | .01 | -.22** | -.12 | -.07 | .05 | .07 | .37*** | .15* |

Note: *n* = 243

^a^- 0=male; 1=female; ^b^- 0= not in a relationship; 1= in a relationship; * = *p* < .05; ** = *p* < .01; *** = *p* < .001
